# Mechanical and Acoustic Performance of Lightweight Cementitious Composites Incorporating Pumice and Expanded Perlite

**DOI:** 10.3390/ma19112274

**Published:** 2026-05-27

**Authors:** Yüksel Furkan Yildirim, Mehmet Emiroğlu

**Affiliations:** 1 Department of Construction, Vocational School, Istanbul Beykent University, Istanbul 34475, Türkiye; 2 Institute of Natural Sciences, Sakarya University, Sakarya 54050, Türkiye; 3 Civil Engineering Department, Faculty of Engineering, Sakarya University, Sakarya 54050, Türkiye; mehmetemiroglu@sakarya.edu.tr

**Keywords:** lightweight cementitious composites, pumice, expanded perlite, acoustic response, sound transmission loss

## Abstract

This study presents a comprehensive experimental investigation of lightweight cementitious composites incorporating pumice and expanded perlite as sustainable substitutes for conventional aggregate systems. Four replacement ratios (25%, 50%, 75%, and 100%) were evaluated to determine their effects on density, compressive strength, flexural strength, modulus of elasticity, and acoustic insulation properties, including the noise reduction coefficient (NRC) and frequency-dependent sound transmission loss (STL). The results showed that increasing the lightweight aggregate content generally reduced the strength-related mechanical properties while improving acoustic performance, particularly in the mid- and high-frequency ranges. Among all mixtures, the expanded perlite-based PRC-1.0 specimen exhibited the best overall acoustic performance, achieving the highest NRC value and the widest STL range. These findings demonstrate a clear trade-off between mechanical strength and acoustic efficiency, indicating that expanded perlite-based lightweight cementitious composites are promising materials for building applications requiring enhanced sound insulation performance.

## 1. Introduction

Concrete is one of the most widely used construction materials because of its affordability, high compressive strength, and durability [1,2,3,4]. However, its relatively high density increases the dead load of structures and may lead to larger structural members and higher construction costs. For this reason, lightweight concrete has attracted increasing attention, particularly in applications where reduced weight, thermal insulation, and acoustic performance are desired [2,5]. Lightweight aggregate concrete (LWAC) is commonly produced by partially or fully replacing conventional aggregates with natural or artificial lightweight aggregates. These porous materials reduce density and may improve thermal and acoustic properties. Common natural lightweight aggregates include pumice, volcanic ash, and slag, whereas expanded clay, expanded glass, and expanded perlite are widely used artificial alternatives [6,7,8].

Previous studies have shown that lightweight aggregates can significantly improve the sound absorption and insulation performance of cementitious composites, particularly through the use of porous aggregates that create internal voids, enhancing acoustic damping, especially at mid- and high-frequency ranges [9,10,11,12,13,14,15,16,17]. However, many previous studies focused mainly on either the mechanical or acoustic performance, while the balance between both properties remains insufficiently understood, as summarized in Table 1.

Therefore, the aim of this study is to experimentally investigate lightweight cementitious composites incorporating pumice and expanded perlite at different replacement ratios. The coupled effects on density, mechanical strength, and acoustic performance were evaluated to identify suitable mixtures for sustainable building applications.

Despite the growing number of studies on lightweight aggregate concretes for acoustic applications, previous research has predominantly focused on single performance indicators, most commonly the sound absorption coefficient (SAC) or noise reduction coefficient (NRC), without simultaneously evaluating mechanical behavior. Therefore, the interaction between porosity development, stiffness reduction, and wave propagation mechanisms remains insufficiently clarified. In particular, the balance between structural requirements and acoustic insulation performance has rarely been quantified, which limits the rational design of multifunctional cementitious composites.

Furthermore, the available literature still lacks a comprehensive methodology capable of systematically relating aggregate type and replacement ratio to both mechanical strength and sound transmission behavior. Accordingly, the present study proposes a coupled mechanical–acoustic evaluation of lightweight cementitious composites containing pumice and expanded perlite. The two aggregate types were directly compared at identical replacement levels of 25%, 50%, 75%, and 100%, while key performance indicators including density, compressive strength, flexural strength, modulus of elasticity, impact resistance, (NRC), and frequency-dependent sound transmission loss (STL) were evaluated. By moving beyond single-parameter analysis, this study provides a practical basis for the development of lightweight cementitious materials with balanced structural and acoustic performance.

## 2. Materials and Methods

### 2.1. Material Properties

**Figure 1:**
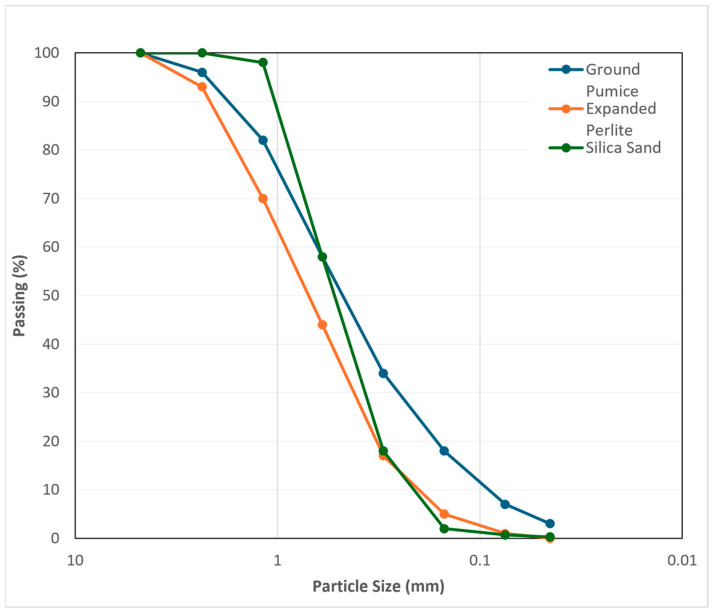
Particle size distribution curves of the lightweight aggregates and silica sand used in this study including ground pumice, expanded perlite, and 30 AFS silica sand.

CEM I 42.5 R type cement was utilized as the binder in this study. The principal chemical components and physical properties of the cement are presented in Table 2. Silica sand, selected for its high-purity silica content and favorable particle size distribution, was employed to enhance the mechanical performance of the concrete and to ensure sufficient compressive strength in lightweight concrete production. The chemical and physical properties of silica sand used in this study are summarized in Table 3. In contrast, lightweight aggregates such as pumice and expanded perlite contribute not only to reducing the unit weight of concrete but also to enhancing its acoustic insulation capability. Expanded perlite was selected in this study due to its low density, porous internal structure, and favorable thermal and acoustic insulation characteristics reported in previous studies. Previous research has demonstrated that expanded perlite-based composites can provide improved sound absorption performance, particularly in the medium- and high-frequency ranges, when combined with cementitious matrices. In addition, expanded perlite has been widely used as a sustainable lightweight aggregate because of its low unit weight and compatibility with cement-based systems [31,32,33]. Pumice aggregate was similarly preferred because of its naturally porous volcanic structure, low density, and potential to improve acoustic insulation while maintaining acceptable mechanical performance [34,35]. The characteristics of these aggregates are summarized in Table 4 and Table 5, respectively. A polycarboxylate-based superplasticizer (BUILDENT WR-780, SAN NOPCO KOREA Ltd., Seoul, Republic of Korea) was incorporated in all mixtures at a fixed dosage of 1% (by weight of cement) to maintain workability, and its properties are listed in Table 6. The particle size distributions of the ground pumice, expanded perlite, and AFS 30 silica sand used in this study were determined by sieve analysis and are presented in Figure 1. The physical and chemical properties of the cement, silica sand, expanded perlite, and superplasticizer were obtained from the manufacturers’ technical datasheets and previous supplier reports. In contrast, the particle size distributions of ground pumice, expanded perlite, and silica sand were experimentally determined through sieve analysis in this study (Figure 1).

### 2.2. Mixture Designs

Concrete batches were prepared using varying proportions of pumice and expanded perlite aggregates. The water-to-cement ratio and the dosage of superplasticizer were kept constant in all formulations to isolate the effects of aggregate substitution. The lightweight aggregates were used as volume replacements for silica sand at four substitution levels: 25%, 50%, 75%, and 100%. Eight distinct mixtures were produced—four containing pumice and four containing expanded perlite. The mixture codes were designated as PMC (pumice-based mixtures) and PRC (perlite-based mixtures), followed by the substitution level (e.g., PMC-0.25, PRC-0.50). Detailed mix proportions are provided in Table 7.

### 2.3. Methods

**Figure 2:**
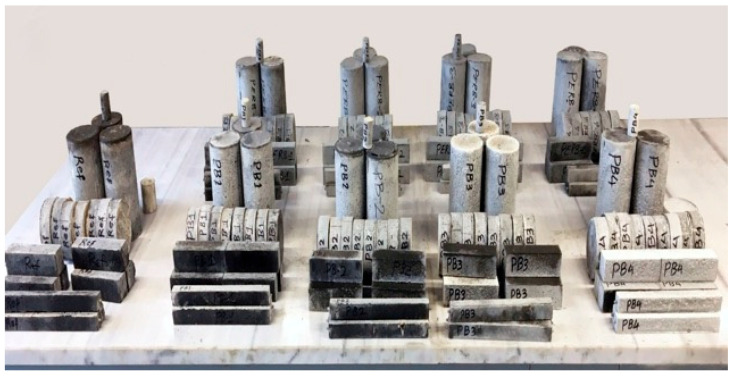
Concrete specimens produced.

Concrete specimens were prepared following the mix proportions detailed in Table 7 and in accordance with TS EN 12390-2 [36]. A polycarboxylate-based high-range water-reducing admixture was incorporated at a dosage of 1% by weight of cement, previously dissolved in 25% of the total mixing water. The dry components (cement and aggregates) were initially mixed with 75% of the total water for 75 s to achieve preliminary homogenization. The remaining water, containing the superplasticizer solution, was then added, and mixing continued at a higher speed for 60 s to ensure full dispersion and uniformity. All specimens were compacted using a vibrating table. To obtain the average, three samples of the same mixture were produced for each test series (Figure 2). The fresh mixtures were cast into molds and cured for 28 days under controlled laboratory conditions.

#### 2.3.1. Fresh Concrete Tests

To assess the consistency and workability of fresh lightweight concrete mixtures, the flow table test was performed in accordance with TS EN 1015-3 [37]. Each measurement was repeated three times to ensure reliability. The spread diameter obtained after the standard flow procedure was recorded and used to evaluate the workability characteristics of the mixtures.

#### 2.3.2. Physical Test Methods

The density of hardened specimens was determined in accordance with TS EN 12390-7 [38]. Water absorption and apparent porosity were evaluated following ASTM C642 [39] based on oven-dry, saturated surface-dry, and submerged mass measurements. All specimens were cured under standard laboratory conditions, and the mechanical and physical tests were performed at curing ages of 7 and 28 days.

#### 2.3.3. Mechanical Test Methods

**Figure 3:**
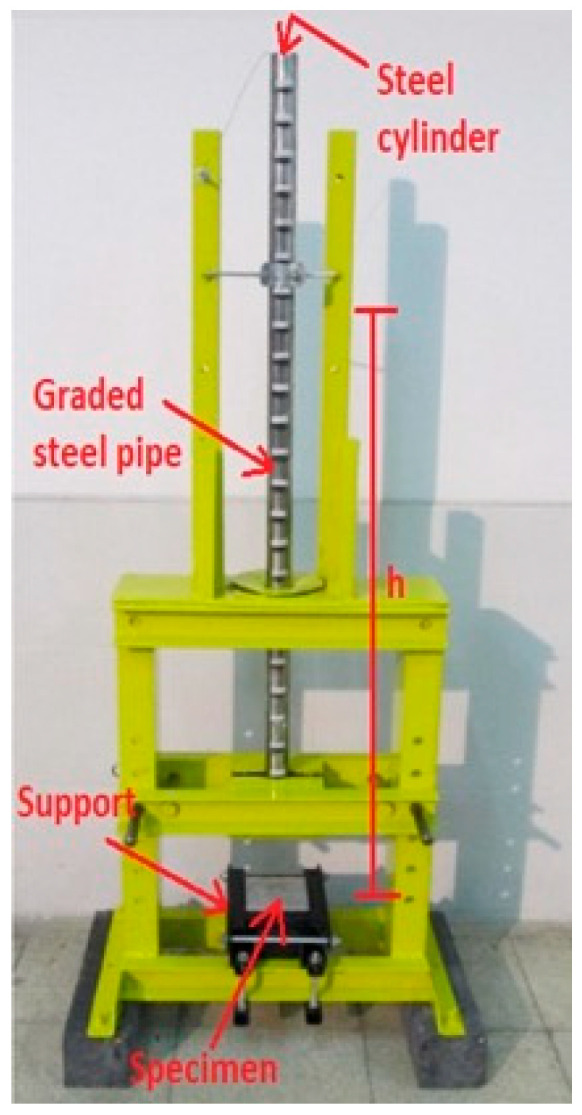
Impact test rig.

**Figure 4:**
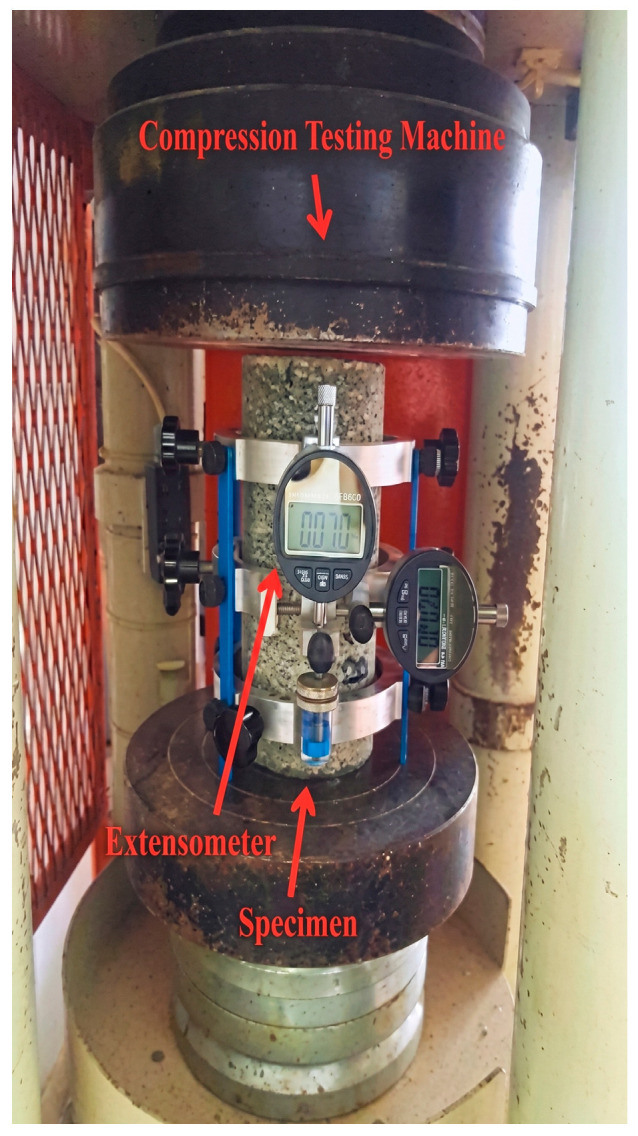
Modulus of elasticity calculation.

**Figure 5:**
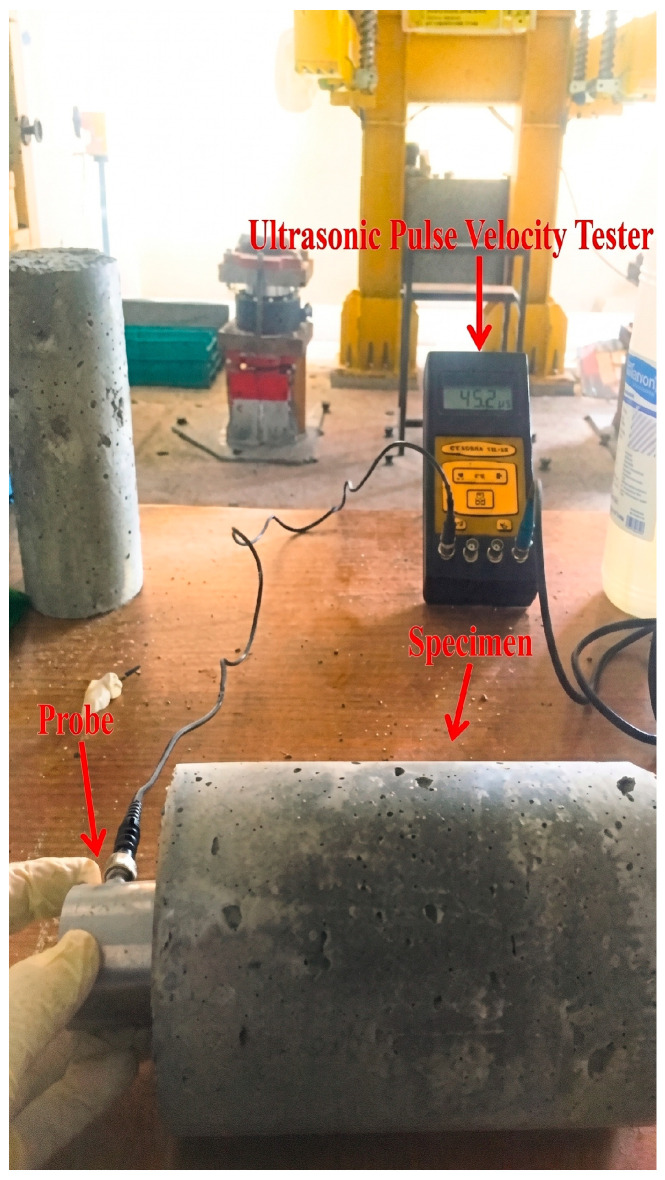
Ultrasonic pulse velocity.

The mechanical properties of the hardened lightweight concrete specimens were evaluated through a comprehensive testing program that included compressive strength, flexural strength, modulus of elasticity, impact resistance, and ultrasonic pulse velocity (UPV) measurements. All tests were performed under controlled laboratory conditions after a standard 28-day curing period, and each measurement was repeated three times (*n* = 3) to ensure statistical reliability. Compressive and flexural strength tests were conducted on prismatic specimens measuring 40 × 40 × 160 mm, prepared in accordance with TS EN 196-1 [40]. The compressive strength was calculated as the maximum load divided by the cross-sectional area, while flexural strength was determined using a three-point bending configuration. The strength per unit weight was calculated with the formula Fc/ρ, where compressive strength is divided by density. Impact resistance was evaluated on cylindrical specimens with dimensions of 100 mm diameter and 30 mm height according to ACI 544.2R-89 [41,42]. A 1.1 kg steel weight was repeatedly dropped from a height of 100 cm until visible cracking or failure occurred. The total number of blows required to cause failure was recorded as the impact resistance index, as shown in Figure 3. Impact energy was calculated based on the potential energy of the falling steel mass using the equation E = n × m × g × h, where n is the number of impacts required to cause failure, m is the hammer mass (1.1 kg), g is the gravitational acceleration, and h is the drop height. The modulus of elasticity was determined using cylindrical specimens of 100 mm diameter and 200 mm height, tested in uniaxial compression according to ASTM C469 [43]. Axial deformation was recorded with two extensometers placed on opposite sides of each specimen, as illustrated in Figure 4. Finally, ultrasonic pulse velocity (UPV) testing was performed on the same cylindrical specimens to assess material uniformity and internal integrity, following ASTM C597 [44,45]. The UPV values, expressed in kilometers per hour (km/h), were obtained by measuring the time required for an ultrasonic pulse to travel through the specimen (Figure 5). According to the standard classification, values exceeding 4.5 km/s indicate concrete with excellent internal quality, while those between 3.5 and 4.5 km/s represent concrete with good internal quality [46,47,48]. UPV measurements were additionally used to evaluate the internal quality, homogeneity, and potential void structure of the lightweight cementitious composites through a non-destructive testing approach.

#### 2.3.4. Acoustic Measurement Methods

**Figure 6:**
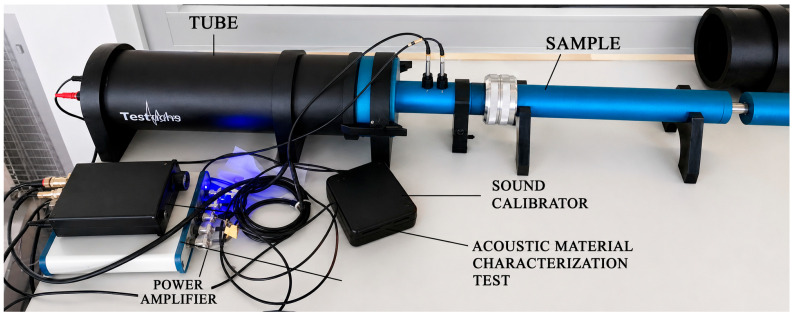
Impedance tube apparatus used for acoustic measurements.

The acoustic performance of the specimens was evaluated using a Brüel & Kjær impedance tube system (Brüel & Kjær, Nærum, Denmark) equipped with a loudspeaker, signal amplifier, and microphones. Measurements were carried out within the frequency range of 50–6300 Hz (Figure 6). The acoustic test specimens were prepared with a diameter of 29 mm and a thickness of 50 mm in accordance with the impedance tube configuration used in the experimental setup. Normal-incidence sound absorption measurements were performed in accordance with ISO 10534-2 [49] using the transfer-function method. For these tests, the microphones were positioned on the source side of the specimen, and the corresponding pressure signals were recorded at two designated microphone positions within the impedance tube.

The (NRC) was calculated as the arithmetic mean of the sound absorption coefficients measured at 250, 500, 1000, and 2000 Hz, in accordance with ASTM C423 [50]:

$$NRC=\frac{\alpha_{250}+\alpha_{500}+\alpha_{1000}+\alpha_{2000}}{4}$$

Following completion of the absorption tests, the impedance tube configuration was rearranged for transmission-related measurements by relocating the microphones to the designated upstream and downstream ports. The transmission performance of the specimens was comparatively evaluated under normal incidence conditions using the pressure-based transfer matrix approach described in ASTM E2611 [51].

The STL-related values were determined from the difference between the incident and transmitted sound pressure levels:

$$STL={Lp}_{i}-{Lp}_{t}$$

where *Lp_i_* represents the incident sound pressure level measured at the source side of the impedance tube, and *Lp_t_* represents the transmitted sound pressure level measured at the receiver side after passing through the specimen.

## 3. Results and Discussion

This section presents the experimental findings obtained from the mechanical and acoustic evaluations of lightweight concrete mixtures produced with varying substitution levels of pumice and expanded perlite aggregates. The results are organized to allow for a direct comparison between the eight mixture groups and cover nine key performance parameters: density, compressive strength, flexural strength, elastic modulus, impact resistance, ultrasonic pulse velocity (UPV), and acoustic performance parameters including SAC, STL, and NRC. All experimental results were evaluated based on the arithmetic mean of three replicate tests conducted for each experiment. The observed experimental trends are interpreted in relation to the mixture design variables and pore structure characteristics.

### 3.1. Fresh Concrete Properties

The fresh-state performance of the mixtures was evaluated using the lightweight concrete table test, and the spread diameters are presented in Table 8.

The results indicate a clear increase in flowability with higher substitution levels of both pumice and expanded perlite. For pumice-based mixtures, replacement ratios of 25%, 50%, 75%, and 100% resulted in spread diameter increases of 10%, 16%, 34%, and 47.2%, respectively, relative to the reference mix. This trend supports previous findings reported by Kurt et al. [52], who attributed the improved workability of pumice concretes to the porous structure of the aggregate, which promotes internal water retention and facilitates lubrication within the mixture, indicating that pumice enhances the workability and flowability of fresh concrete due to its inherently porous structure. Consistent with Kurt et al. [53], the porous nature of pumice improves internal water retention and enhances mixture lubrication, increasing workability. Nevertheless, excessive flowability can reduce stability and segregation resistance, as highlighted by Hamasalh et al. [54], making a proper balance essential.

A similar but significantly more pronounced effect was observed for expanded perlite. The spread diameters increased by 127%, 140%, 158%, and 168% at the same substitution levels, confirming the strong influence of perlite on flowability. Hamidi [55] also reported an 83% increase in the spread diameter for mixtures containing 25% expanded perlite, consistent with the present results. The superior contribution of perlite to workability is primarily associated with its highly porous microstructure and pronounced hydrophilicity, which enable the aggregate to release absorbed moisture during mixing. This mechanism reduces viscosity and enhances the overall plasticity of the mixture [53]. The relatively higher spread values observed in the present study compared with some previous studies may be associated with differences in aggregate grading, water retention behavior, superplasticizer dosage, and the highly porous structure of the lightweight aggregates used. Nevertheless, excessive fluidity may compromise the stability and segregation resistance of the mixture. Consequently, a hybrid mix design incorporating both pumice and expanded perlite may offer optimal performance by effectively balancing the rheological properties of fresh concrete.

### 3.2. Hardened Concrete Properties

#### 3.2.1. Physical Characteristics of Hardened Concrete

**Figure 7:**
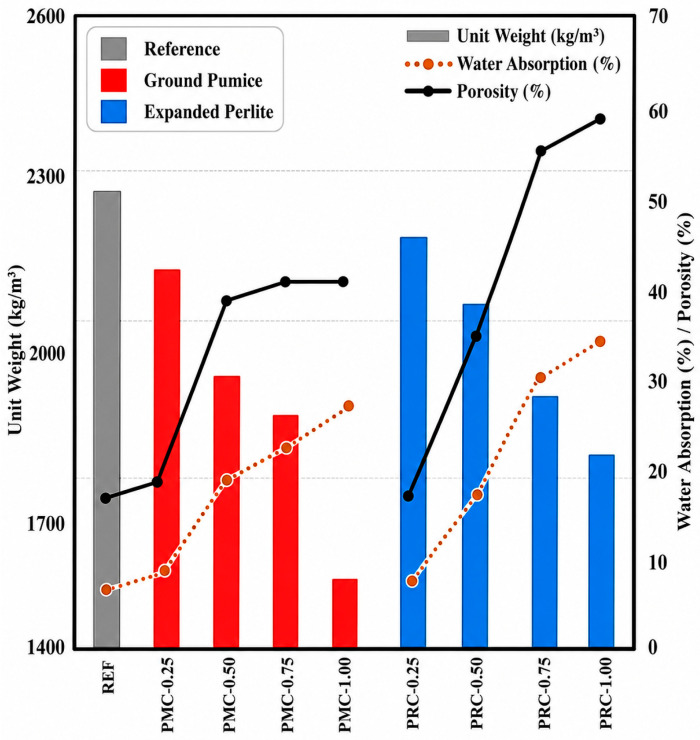
Unit volume weight, water absorption rate, and porosity of the mixtures.

Figure 7 presents the unit volume weight, water absorption rates, and porosity of the hardened concrete specimens.

As expected, the incorporation of lightweight aggregates significantly reduced the density of the mixtures. The reference mixture exhibited a unit volume weight of 2281.25 kg/m^3^, whereas the PMC-1.0 (100% pumice) mixture showed a 35% reduction, and the PRC-1.0 (100% expanded perlite) mixture showed a 23% reduction. This reduction is directly attributable to the highly porous internal structure of pumice and expanded perlite.

The water absorption rates followed a similar trend. The reference sample displayed an absorption rate of 7.0%, which increased to 27.5% for the fully pumice-based PMC-1.0 mix and to 34.15% for the fully perlite-based PRC-1.0 mix. When porosity values are examined, the reference mixture exhibited a porosity of 15.97%, whereas the porosity increased markedly with higher lightweight aggregate content. In the pumice series, porosity rose from 18.04% (PMC-0.25) to 41.14% (PMC-1.0). In the expanded perlite series, the increase was even more pronounced, ranging from 14.87% (PMC-0.25) to 60.12% (PMC-1.0). These results indicate that both pumice and expanded perlite increased the pore volume and water absorption capacity of the mixtures. The effect was more pronounced in the expanded perlite series due to its finer and more interconnected pore structure. Lanzón and Ruiz [56] reported that the extensive pore network of expanded perlite leads to markedly higher water absorption, establishing a strong inverse relationship between density and absorption capacity. Likewise, Esfandiari et al. [57] observed that the water absorption rate increased from 5% to 9% as the perlite substitution rose from 10% to 20%, further confirming that absorption is strongly dependent on aggregate porosity. Bakhishi et al. [58] also demonstrated that replacing conventional aggregates with 55–100% expanded perlite resulted in a 20% reduction in unit weight. Similarly, Muhtar [59] reported reductions of 15%, 27%, 30%, and 32% in unit weight for mixtures containing 25%, 50%, 75%, and 100% pumice, respectively. These results collectively confirm that increasing the proportion of lightweight aggregates reduces the density while substantially increasing the water absorption. Beyond the physical implications, reductions in density are known to improve the seismic performance of structural systems by lowering mass, while increased porosity contributes positively to thermal and acoustic insulation due to reduced solid-phase continuity.

#### 3.2.2. Compressive Strength

**Figure 8:**
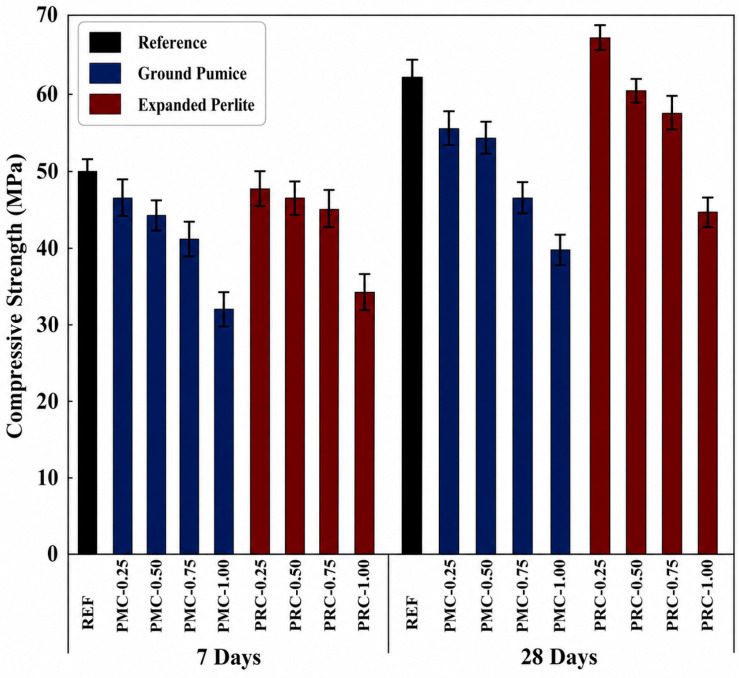
Compressive strength of REF-, pumice (PMC)-, and perlite (PRC)-based mixtures cured for 7 and 28 days.

Compressive strength tests were conducted on the specimens at curing ages of 7 and 28 days. The results obtained at these curing ages are illustrated in Figure 8.

In the tests, the 28-day compressive strength of the reference specimen was determined as 60.12 MPa. In the concrete series produced with pumice, the compressive strength decreased with the increase in pumice ratio and was measured as 40.47 MPa, with a 33% decrease in the PMC-1.0 series. In the expanded perlite-substituted concrete series, the 25% expanded perlite-substituted concrete series showed an increase of 11% and was measured as 67.18 MPa. The relatively higher compressive and flexural strength observed in the PRC-0.25 mixture may be associated with improved particle packing and filler effects at low replacement levels. The fine porous structure of expanded perlite may have contributed to a more homogeneous internal matrix and improved stress distribution within the composite. In addition, limited internal curing effects caused by the water absorption capacity of lightweight aggregates may also have contributed to the observed mechanical performance at low substitution ratios. In the PRC-1.0 series containing 100% expanded perlite, the strength decreased by 21% compared to the reference specimen and was determined as 47.18 MPa. Across both lightweight aggregate series (PMC and PRC), 28-day compressive strengths increased by 20% on average compared to the 7-day compressive strengths of the specimen age. It is known in the literature that changing the Portland cement ratio at high rates leads to a decrease in the amount and alkalinity of the hydration product at early ages. However, it has been reported that ground pumice aggregate may contribute to improved compressive strength development at later ages [52].

Pumice aggregate is generally used in concretes with sound and thermal insulation properties, but it is known to have negative effects on strength, as reported in the literature. Gunduz [60] investigated the influence of varying pumice aggregate-to-cement ratios on the mechanical properties of low-strength concrete. According to the results of the study, it was observed that the compressive strength decreased from 15 MPa to 2 MPa with the increase in the pumice/cement ratio from 6 to 30. However, the unit volume weight of the mixture with a pumice/cement ratio of 30 decreased by approximately four times compared to the mixture with a ratio of 6, enabling the achievement of the expected insulation performance of the material produced. Hossain [61] investigated the effects of substituting different proportions of pumice powder in lightweight concretes. Considering compressive strengths, lightweight concretes containing 50% pumice powder showed more than a 50% reduction in 28-day strengths. High amounts of pumice in cementitious composites significantly retard the hydration process, leading to reduced formation of calcium silicate hydrate (C-S-H) and calcium aluminate hydrate (C-A-H) gels [62], which can lead to reductions in strength.

Previous studies have also reported similar trends regarding the influence of expanded perlite on compressive strength development. Expanded perlite may exhibit pozzolanic behavior on cementitious materials and may have positive effects on strength and durability at advanced ages. Bakhshi [58] used expanded perlite in varying proportions as a replacement for conventional aggregate and observed a decrease in compressive strength with increasing perlite content, attributed to its highly porous structure and low mechanical strength. Esfandiari and Loghmani [57] investigated the effect of perlite dust and silica fume on compressive strength and the microstructural characterization of self-compacting concrete with lime-cement binder. According to the results, a 6% positive effect of perlite powder on compressive strength at advanced ages was observed. However, high perlite content prevented the development of C-S-H gel, leading to a decrease in the compressive strength of the specimens at 28 and 90 days of age. In the present study, the mixture containing 25% perlite exhibited an 11% increase in 28-day compressive strength, which may be attributed to the pozzolanic properties of this mineral material. Lanzon and Ruiz [56] investigated the effects of expanded perlite on lightweight concretes. The experimental results showed that expanded perlite contents above 1.77% adversely affected the mechanical strength, absorbency, and water absorption, while workability and water retention were generally improved.

#### 3.2.3. Specific Strength

**Figure 9:**
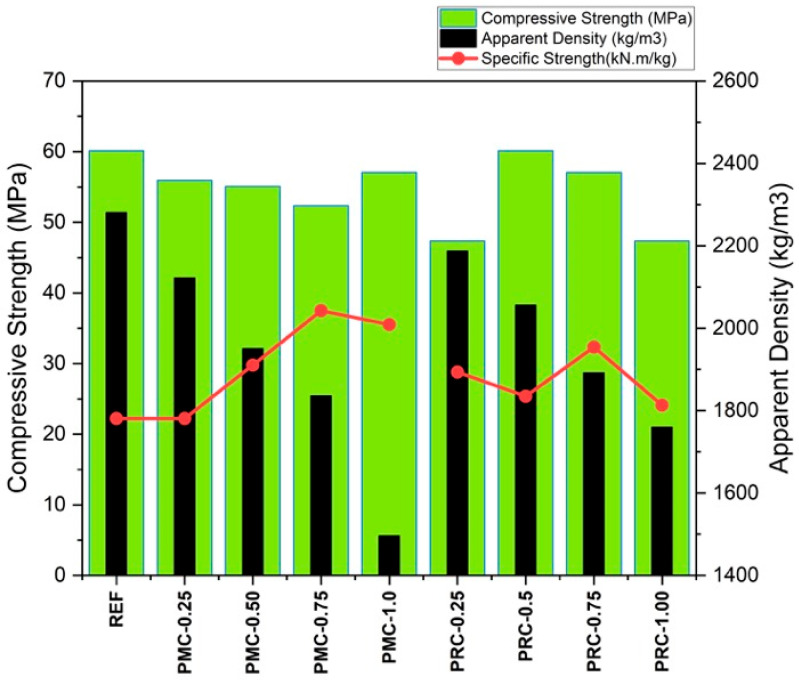
Compressive strength and apparent density of the concrete mixtures.

Specific strength values were calculated by dividing the compressive strength of each concrete mixture by its corresponding unit weight, and the results are presented in Figure 9.

The specific strength of concrete, defined as the compressive strength per unit mass, serves as a vital metric for evaluating material efficiency in structural applications. In this study, the incorporation of pumice and expanded perlite significantly enhanced the specific strength of concrete. The reference mixture exhibited a specific strength of 26.35 kN·m/kg. Among the pumice-modified series, the PMC-0.75 sample displayed the highest value at 30.71 kN·m/kg, representing a 24% improvement. Similarly, the perlite-substituted samples demonstrated increased specific strength with higher substitution rates, with the PRC-0.75 series achieving 29.24 kN·m/kg, corresponding to an 11% increase. These results indicate that a 75% substitution level yields optimal enhancement for both materials, whereas even full substitution (100%) maintains a comparable specific strength performance relative to the reference mixture.

Previous studies have investigated the relationship between aggregate type, density, and compressive strength in order to further optimize specific strength. Sharma et al. [63] reported that complete substitution with expanded perlite resulted in a 24% reduction in specific strength, despite a notable decrease in density. Numan et al. [64] observed lower specific strengths in perlite-modified concretes compared to those with pumice, aligning with our findings [63]. Chen et al. [65] demonstrated the positive impacts of basalt fiber and recycled aggregates on specific strength, revealing increases of up to 49%. These comparisons underscore that while lightweight aggregates contribute to improved specific strength, the type of aggregate, the application of admixtures, and the overall mixture design play critical roles in influencing the final outcome. Therefore, the integration of lightweight materials with fibers or chemical admixtures appears to be a promising strategy for the development of high-performance, lightweight concrete.

#### 3.2.4. Flexural Strength

**Figure 10:**
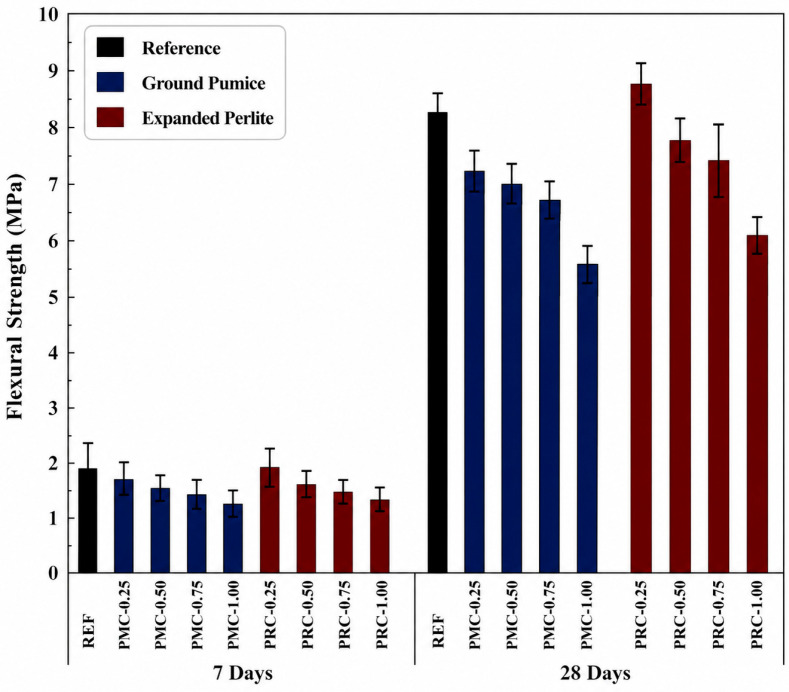
Results of the 7-day and 28-day flexural strength tests.

Flexural strength tests were performed at 7 and 28 days on specimens prepared in accordance with TS EN 196-1 [40]. For each mixture, three replicate specimens (*n* = 3) were tested to ensure statistical reliability. The results are presented in Figure 10.

After 28 days of curing, the reference mixture exhibited a flexural strength of 8.27 MPa. For pumice-based mixtures, the lowest value was recorded for the PMC-1.0 mixture, which reached 5.30 MPa, corresponding to a 36% reduction compared with the reference specimen. This decrease is attributed to the elevated porosity and weaker interfacial bonding associated with fully pumice-based matrices.

In contrast, mixtures incorporating expanded perlite exhibited a different trend. The PRC-0.25 mixture achieved the highest flexural strength among all lightweight aggregate mixtures, reaching 8.73 MPa, which reflects a 5.5% improvement over the reference. This enhancement suggests that limited perlite substitution can improve crack-bridging behavior and stress distribution due to favorable particle morphology at low replacement levels. However, the flexural strength decreased as the substitution level increased; PRC-1.0 exhibited a strength of 6.15 MPa, indicating that excessive porosity at high perlite contents adversely affects crack resistance and flexural performance.

Pumice, characterized by its volcanic origin and cellular structure, contributes to weight reduction; however, it may adversely affect mechanical strength by limiting particle interlock and bond development at the interface [66]. Conversely, expanded perlite, recognized for its low density and thermal insulation properties, is highly porous, which can introduce voids and compromise the integrity of the internal matrix [67]. The observed reduction in flexural strength in mixtures with high levels of substitution can be attributed to alterations in the interfacial transition zone (ITZ) between aggregate particles and cement paste. Pumice and perlite possess distinct surface textures and chemical properties compared to conventional aggregates, which may hinder bond formation within the ITZ [66,67,68]. A compromised ITZ facilitates the initiation and propagation of microcracks under flexural loading, subsequently reducing the overall structural performance. Furthermore, the lower elastic modulus associated with lightweight concretes leads to greater deformation under stress, further diminishing flexural strength [68]. These factors elucidate the trends identified in this study, where moderate levels of perlite substitution enhanced performance, while elevated replacement ratios resulted in a reduction in flexural capacity.

Specifically, the lower performance of the PMC-1.0 mixture (5.30 MPa) can be attributed to a decrease in mechanical properties due to increased porosity and a compromised interfacial bond caused by the inherent voids of pumice [69].

**Figure 11:**
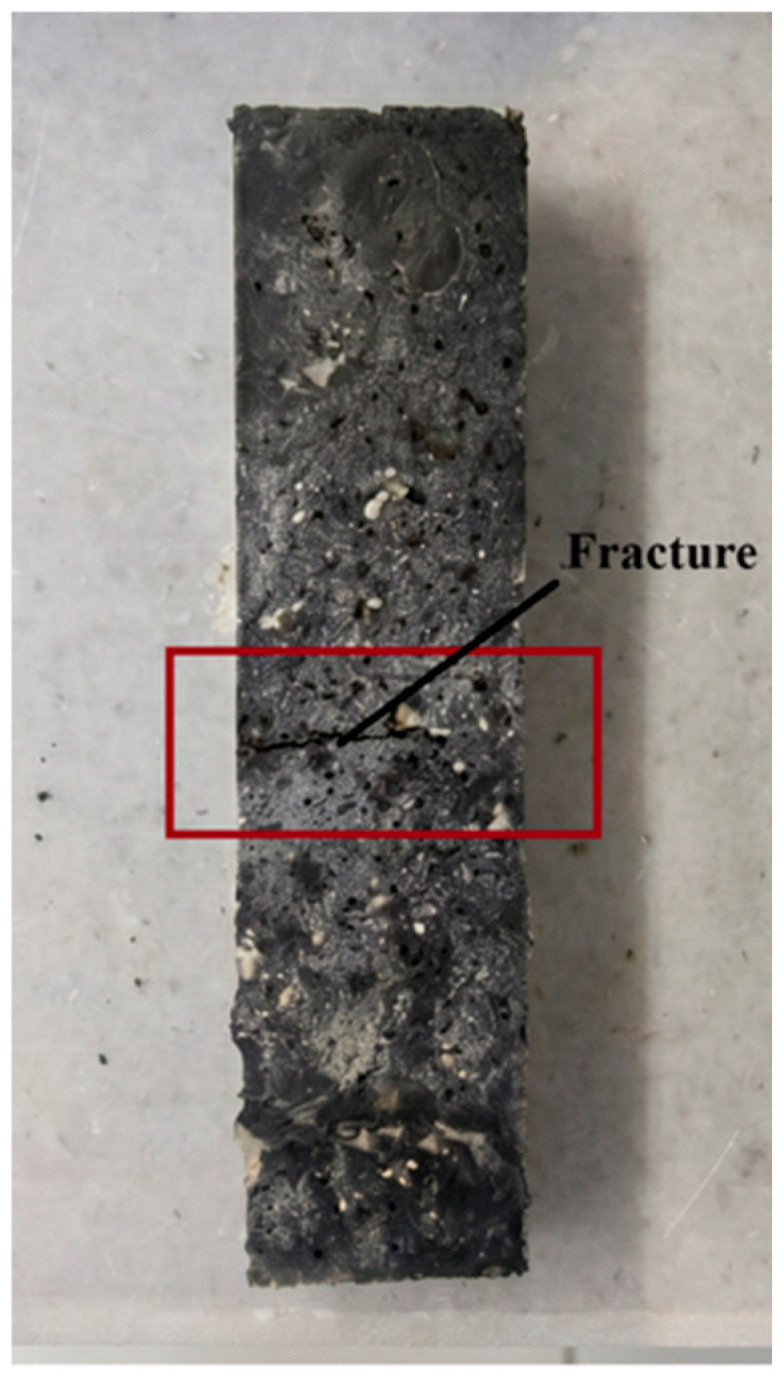
Representative flexural fracture pattern observed in the lightweight cementitious composite specimen after mechanical testing.

In contrast, the enhanced flexural strength of PRC-0.25 (8.73 MPa) suggests that limited perlite inclusion optimizes the microstructural arrangement, thus improving crack-bridging capabilities. As documented by Behera et al., the presence of favorable particle morphology at lower replacement levels significantly benefits stress distribution [70]. However, the subsequent drop in performance at higher perlite levels indicates that excessive porosity disrupts connective matrix structures, which aligns with conclusions by Yan regarding the detrimental effects of voids on strength [71]. A representative flexural fracture pattern observed after mechanical testing is presented in Figure 11.

#### 3.2.5. Impact Test

**Figure 12:**
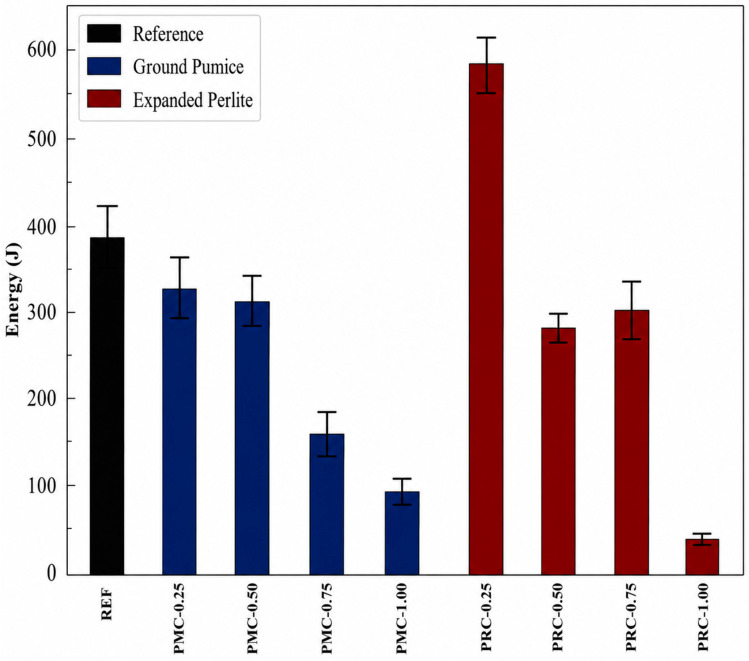
Impact test results of the concrete mixtures.

**Figure 13:**
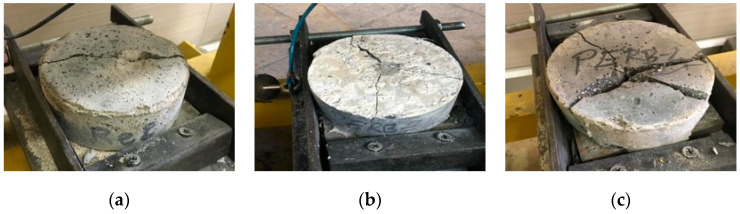
Impact test specimens of (**a**) reference specimen, (**b**) 75% expanded perlite-substituted PRC-0.75, and (**c**) 100% perlite-substituted PRC-1.0 specimens.

Impact resistance was evaluated by dropping a 1.1 kg steel mass from a height of 120 cm onto the specimens and recording the total impact energy required to induce visible cracking or failure. For each mixture, three replicate specimens (*n* = 3) were tested to ensure measurement consistency. The impact strength results for all mixtures are presented in Figure 12, while Figure 13 shows the specimens after testing.

The highest impact energy was observed in the mixture containing 25% expanded perlite, indicating that limited perlite substitution enhances the energy absorption capacity of the composite. However, the impact resistance decreased progressively with higher perlite contents, reaching its lowest value in the mixture with 100% expanded perlite. This reduction is attributed to the inherently weak mechanical properties and fragile pore structure of expanded perlite, which reduce the material’s ability to resist crack initiation and propagation at high substitution ratios [58]. The PRC-0.50 and PRC-0.75 mixtures exhibited similar impact resistance values, although PRC-0.75 showed slightly higher energy absorption. This behavior may be associated with increased internal porosity, which enhances the energy dissipation capacity but can also weaken the structural integrity of the composite at higher replacement levels.

In the pumice-substituted series, the increase in pumice ratio decreased the impact strength; while the energy absorption capacity of the reference specimen was 400 J, this value was determined as 100 J in the 100% pumice-substituted specimen. Similar findings in the literature indicate that the use of lightweight aggregates leads to a reduction in the impact strength of concrete [72]. Heever et al. [73] reported that concretes containing more than 50% pumice became more brittle in terms of performance and cracking behavior; Hariyadi and Tamai [74] observed a decrease in mechanical strength in concrete series using pumice instead of sand and stated that these concretes could be used as impact absorbers due to their high strain capacity. Similar research emphasizes how increased internal voids negatively affect concrete’s ability to resist crack propagation, particularly at elevated substitution ratios [75]. Additionally, the studies by Ali et al. corroborate that lightweight aggregates like pumice and perlite compromise impact strength due to their weak mechanical properties, although they can contribute to enhanced energy absorption [76].

#### 3.2.6. Modulus of Elasticity and Ultrasonic Sound Velocity Determination

**Figure 14:**
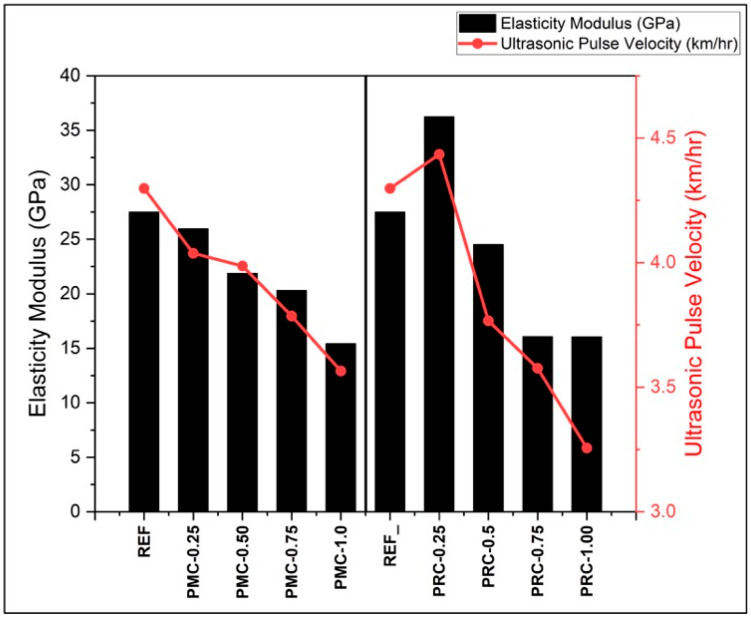
Comparison of ultrasonic pulse velocity and modulus of elasticity.

The modulus of elasticity was determined on 100 × 200 mm cylindrical specimens following the procedures specified in ASTM C469 [43]. Likewise, ultrasonic pulse velocity (UPV) measurements were conducted on the same specimen geometry in accordance with ASTM C597-22 [44]. For each mixture, three replicate specimens (*n* = 3) were tested to ensure the reliability of the results. The UPV values and the modulus of elasticity results are shown in Figure 14.

According to the experimental findings, the highest modulus of elasticity was obtained in the mixture incorporating 25% expanded perlite, reaching 36,000 MPa, which corresponds to a 33% increase relative to the reference mixture. This improvement at low substitution levels may be attributed to the filler-like behavior of perlite particles, which enhance stiffness by improving particle packing and facilitating better stress transfer within the cementitious matrix [77]. As the expanded perlite substitution ratio increased, the modulus of elasticity decreased substantially. In PRC-1.0, the elastic modulus dropped to 15,000 MPa, representing a 45% reduction compared to the reference. The decline at higher replacement levels is attributed to the significantly higher porosity of perlite, which weakens the aggregate–matrix interface and reduces the material’s ability to resist elastic deformation. These results indicate that while limited perlite substitution may enhance stiffness, excessive incorporation adversely affects the elastic response due to the dominance of weak, highly porous aggregate phases.

Kapeluszna et al. [78] reported that using 20% expanded perlite improved the mechanical properties due to lower water demand. However, as the expanded perlite content increased, the modulus of elasticity decreased because of higher water demand. Suseno [79] measured modulus of elasticity values of 22.46 GPa, 16.67 GPa, and 17.83 GPa for specimens made with coarse aggregate, pumice, and scoria, respectively. The observed reduction in elasticity was attributed to the stronger interfacial bond between coarse aggregates and the cement paste, as well as the lower porosity of pumice and scoria aggregates. Similarly, Hossain et al. [80] reported a modulus of elasticity of 18.2 GPa in the reference specimens, which decreased by 43% to 10.5 GPa with 100% pumice substitution. The reference specimen in this study exhibited a modulus of elasticity of 27,000 MPa. Increasing the pumice substitution ratio led to a substantial decline, reaching 15,000 MPa at 100% substitution, representing a 45% reduction. These findings are consistent with previous studies reporting reductions in elastic modulus with increasing pumice content [81]. Sarıdemir [82] similarly noted that elasticity decreased with increasing pumice content, while Muhtar [59] reported reductions of 19%, 37%, 44%, and 51% in elasticity for substitution rates of 25%, 50%, 75%, and 100%, respectively. Chand et al. [83] observed a 10% decrease with as little as 4% pumice substitution.

Analysis of the ultrasonic pulse velocity (UPV) results presented in Figure 14 revealed that the UPV of the reference and PRC-0.25 series—both demonstrating the highest mechanical strengths—was the highest among all mixtures. In mixtures with increasing amounts of lightweight aggregate, a decrease in UPV was recorded, attributed to the high void content of the aggregates. While the reference sample exhibited a UPV of 4.25 km/s, this value declined by 24% to 3.20 km/s in the PRC-1.0 series with 100% expanded perlite substitution.

Cement content significantly influenced the UPV values of the concrete mixtures; variations in porosity and density among the aggregates also contributed to differences in UPV [84]. Mohammed et al. [85] found that the type of aggregate significantly impacted the UPV, with lightweight and highly absorbent aggregates reducing the velocity. Temiz et al. [86] similarly reported a 70% reduction in UPV in mixtures with 30% pumice substitution compared to the reference. A comparison of ultrasonic velocity and modulus of elasticity is illustrated in Figure 13. The results for the PRC-0.25 series revealed high values for both parameters, suggesting a dense, well-bonded internal structure with strong mechanical and acoustic performance. In contrast, the PMC-1.0 and PRC-1.0 series exhibited lower values for both modulus of elasticity and UPV, indicating weaker mechanical properties and higher porosity. Mixtures with 25%, 50%, and 75% substitutions of pumice and expanded perlite showed intermediate values, reflecting a more balanced profile in terms of porosity and density. The graph illustrates a general positive correlation between the modulus of elasticity and ultrasonic wave velocity. This correlation implies that as the mechanical strength increases, the material becomes more compact and transmits sound waves more efficiently, highlighting the influence of porosity and bond quality on both UPV and mechanical strength.

#### 3.2.7. Sound Absorption Coefficient (SAC)

**Figure 15:**
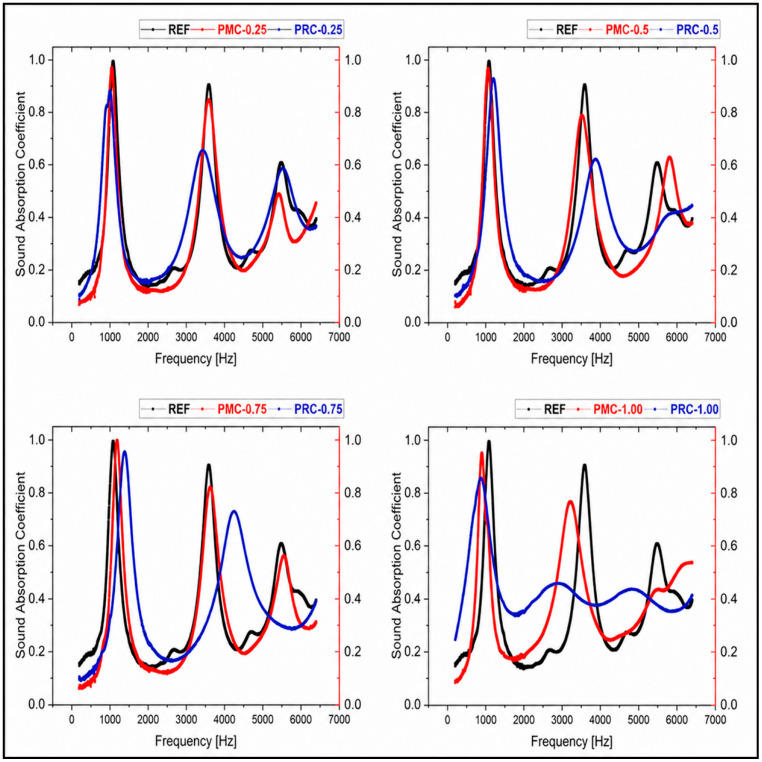
Sound absorption coefficient of lightweight concretes containing pumice and expanded perlite measured using an impedance tube.

The sound absorption coefficients of the mixtures were measured using the impedance tube method, and the results are presented in Figure 15. The sound absorption coefficient represents the ratio of absorbed sound energy to the incident sound energy on the material surface. For clarity of comparison, the pumice- and expanded perlite-based mixtures with identical substitution ratios are plotted together, while the reference mixture is shown separately. This representation enables a direct evaluation of the influence of the lightweight aggregate type and replacement level on the acoustic absorption.

Theoretically, when sound waves interact with a concrete surface, three primary mechanisms occur depending on the internal pore structure: part of the sound is transmitted, part is reflected, and part is absorbed within the material matrix [87]. Echeverria et al. [88] emphasized that the sound absorption coefficient is strongly governed by the permeability and interconnected porosity of the material. In the present study, all pumice-replaced mixtures exhibited sound absorption curves similar in shape to that of the reference specimen, although their amplitudes varied with increasing substitution levels. The mixture with 25% pumice showed behavior comparable to the reference, while higher replacement levels shifted the absorption peaks across different frequencies, indicating frequency-dependent changes in pore–wave interactions. The higher absorption peaks observed in the reference specimen at certain frequencies may be associated with its denser and more continuous matrix structure, which can promote stronger surface reflection and resonance effects within specific frequency ranges. In contrast, the incorporation of lightweight aggregates altered the internal pore distribution and shifted the absorption behavior over a broader frequency spectrum.

For pumice-replaced mixtures, the SAC reached values as high as 1.0 at 1100 Hz and 0.95 at 3500 Hz. Notably, the mixture containing 100% pumice maintained SAC values above 0.5 throughout the 2500–4500 Hz range, demonstrating strong absorption performance at medium and high frequencies. These observations align with Bozkurt et al. [89], who reported significant improvements in sound absorption between 1400–2700 Hz when river sand was replaced with pumice, as well as Soyaslan [90], who found enhanced absorption in the 500–1100 Hz range for mixtures containing moderate pumice substitution.

Compared with the pumice-replaced mixtures, the expanded perlite-replaced mixtures displayed distinctly different acoustic behaviors. The 25% expanded perlite mixture exhibited lower peak SAC values than the reference specimen; however, it provided sound absorption over a broader frequency range. This behavior may be attributed to the highly porous internal structure of expanded perlite, which promotes wider frequency-dependent absorption characteristics. The PRC-0.50 and PRC-0.75 mixtures demonstrated similar performance below 2000 Hz, but above 3000 Hz, the PRC-0.75 exhibited sound absorption behavior over a relatively wider frequency range, which may be associated with increased pore continuity. The PRC-1.0 mixture, containing 100% expanded perlite, reached SAC values of approximately 0.5 at frequencies above 2000 Hz, representing the highest overall sound absorption capacity among all mixtures tested. These findings are in agreement with Li et al. [91], who reported that perlite substitution shifts the absorption behavior toward lower frequencies while enhancing overall performance, and Benjeddou et al. [33], who demonstrated a 26% increase in sound reduction at 1500 Hz for mixtures with 50% perlite.

#### 3.2.8. Noise Reduction Coefficient (NRC)

**Figure 16:**
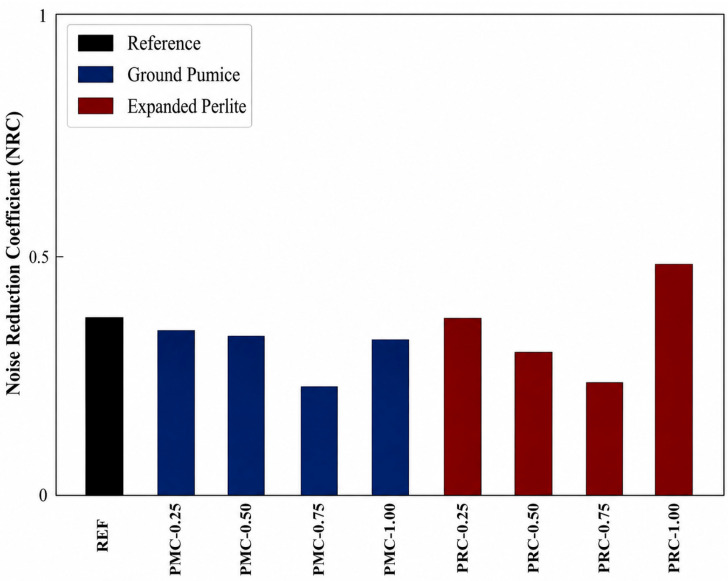
NRC values of the concrete mixtures.

The noise reduction coefficient (NRC) values were calculated for each mixture based on the impedance tube measurements, and the results are presented in Figure 16.

While the NRC values of the reference specimen were relatively low, the mixtures containing pumice and expanded perlite mixtures exhibited improved NRC performance at higher replacement levels, particularly in the PRC-1.0 mixture. Among all specimens, the PRC-1.0 mixture achieved the highest NRC value, indicating improved sound absorption over a broader frequency range.

The reference and pumice-based mixtures showed relatively similar NRC values below 2000 Hz, whereas the PRC-0.50 and PRC-0.75 mixtures demonstrated improved performance, which may be associated with their pore structure and the acoustic contribution of the lightweight aggregate phase, particularly at lower frequencies.

At lower replacement levels (25%), both the pumice- and perlite-based mixtures exhibited acoustic behavior close to that of the reference mixture. However, as the replacement ratio increased, a clearer improvement in NRC was observed. In particular, the expanded perlite series showed a more consistent increasing trend, with the PRC-1.0 specimen providing the highest overall NRC performance.

Overall, the results indicate that expanded perlite was more effective than pumice in enhancing the NRC values of the tested composites, which may be related to its lower density and more favorable internal pore distribution. The observed acoustic improvements can be primarily attributed to the increased porosity and internal void structure introduced by the lightweight aggregates. The porous morphology of pumice and expanded perlite promotes multiple sound wave reflections and energy dissipation within the cementitious matrix, thereby enhancing sound absorption behavior. However, the increase in pore volume and the relatively weaker interfacial bonding between the lightweight aggregates and the cement paste may also contribute to reductions in compressive and flexural strength. Therefore, the experimental results demonstrate the inherent trade-off between mechanical resistance and acoustic efficiency in lightweight cementitious composites. Although direct microstructural characterization techniques such as SEM or pore structure imaging were not employed in the present study, the observed mechanical and acoustic behaviors are consistent with the porous internal morphology typically reported for pumice and expanded perlite aggregates in the literature. Previous studies have shown that the increased pore connectivity and internal void distribution of lightweight aggregates can enhance sound wave dissipation while simultaneously reducing mechanical strength due to weaker aggregate–paste interactions, the relatively weak interfacial transition zone (ITZ), and increased porosity within the cementitious matrix [23,92].

**Figure 17:**
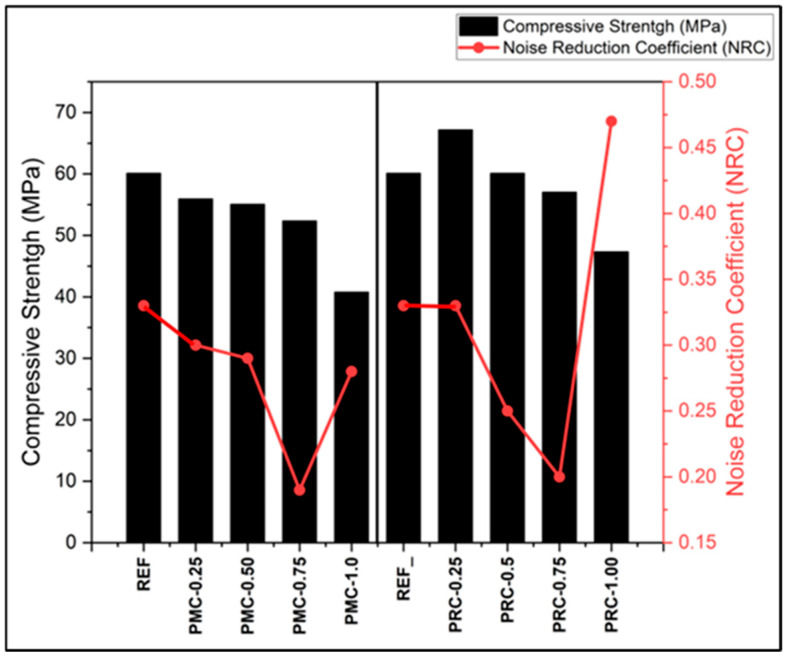
Relationship between compressive strength and NRC of the produced lightweight cementitious composites.

Tie et al. [10] reported that cement-based materials with improved sound absorption performance generally tend to exhibit lower density values. A similar trend was observed in the present study, where increasing the lightweight aggregate content resulted in improved NRC values. A comparison between the compressive strength and NRC values of the produced samples is presented in Figure 17.

The results indicate a trade-off between mechanical strength and acoustic performance in lightweight cementitious composites. In the pumice-based series, increasing replacement ratios generally reduced the compressive strength, while the NRC values did not increase proportionally. In contrast, the expanded perlite series exhibited improved acoustic performance at higher replacement levels, particularly in the PRC-1.0 mixture, although this was accompanied by reductions in compressive strength. These findings suggest that the acoustic response of the mixtures is strongly influenced by pore structure, density, and internal connectivity of the lightweight aggregates.

Similar findings have been reported in previous studies [93,94], where mixtures with lower density and higher pore connectivity exhibited superior NRC performance.

Overall, the expanded perlite series, particularly PRC-1.0, provided the most favorable balance between acoustic efficiency and mechanical performance, whereas the reference and low-replacement mixtures maintained higher compressive strength. Comparable relationships between porosity, sound absorption, and strength reduction have also been reported for cementitious composites containing pumice and perlite aggregates [95,96,97].

#### 3.2.9. Frequency-Dependent Sound Transmission Loss (STL)

**Figure 18:**
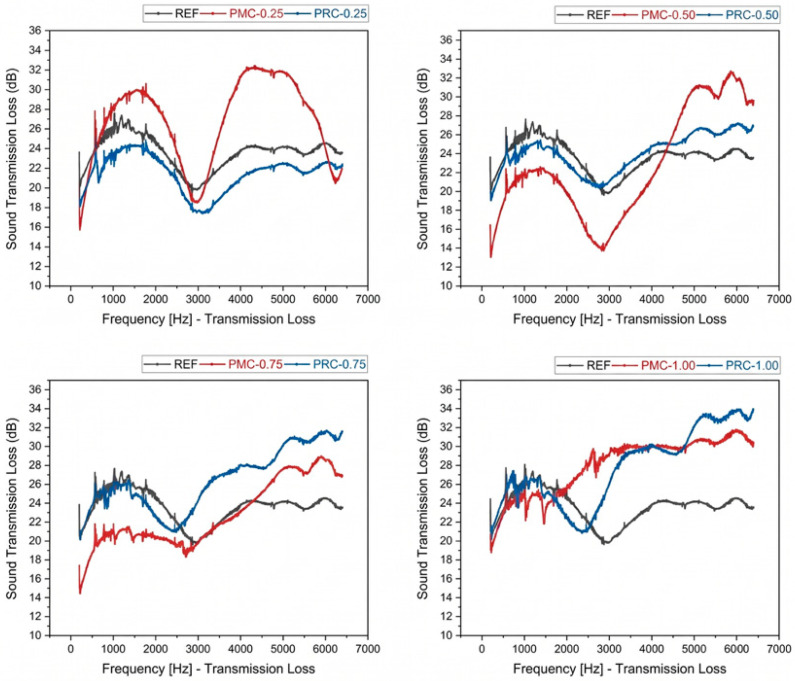
Frequency-dependent sound transmission loss (STL) curves of the lightweight cementitious composites incorporating pumice and expanded perlite aggregates measured using an impedance tube setup.

The STL values measured using the impedance tube are presented in Figure 18.

The frequency-dependent STL results of the produced lightweight cementitious composites are presented in Figure 18.

In the pumice-based series, the STL behavior varied depending on the replacement ratio and frequency range. At lower replacement levels, the STL curves remained relatively close to that of the reference mixture, whereas higher pumice contents generally resulted in increased attenuation in the mid- and high-frequency regions. In particular, the PMC-0.75 and PMC-1.0 mixtures exhibited higher STL-related values above 3500 Hz.

A similar trend was observed in the expanded perlite series. The perlite-based mixtures showed a more stable and gradually increasing STL response with increasing frequency. Among all specimens, the PRC-1.0 mixture exhibited the highest STL-related values in the high-frequency region, reaching values above 30 dB. These results suggest that the incorporation of lightweight aggregates influences transmission behavior through changes in density, pore structure, and internal wave dissipation mechanisms. In particular, the expanded perlite series demonstrated more effective attenuation at higher frequencies, which may be associated with its more uniform pore distribution and lower density. The frequency-dependent acoustic behavior observed in the lightweight cementitious composites may be associated with differences in pore structure, internal void connectivity, and sound wave interaction mechanisms at different frequency ranges. At higher frequencies, the porous internal structure of lightweight aggregates can enhance multiple wave reflections and viscous energy dissipation, leading to improved sound absorption and transmission loss performance.

Overall, the incorporation of pumice and expanded perlite generally improved the frequency-dependent sound transmission loss behavior of the tested composites, with more pronounced effects observed in the high-frequency range. Suardana et al. [98] reported that STL values were lowest at 125 Hz, increased and peaked around 500 Hz, and then fluctuated up to 4000 Hz. They also noted that mixtures containing higher proportions of pumice achieved superior STL performance compared to gypsum-based counterparts. Similarly, Tan et al. [99] examined the STL characteristics of low-density and porous construction materials and reported that STL values generally peaked within the 3000–5500 Hz frequency range, while lower frequencies exhibited more stable yet lower STL performance. According to STL classification across the low-, mid-, and high-frequency zones, material stiffness (rigidity) plays a dominant role in sound insulation at low frequencies [100]. Cement-based composites incorporating pumice and expanded perlite possess relatively high surface stiffness and density compared with many other porous construction materials. As a result, these mixtures tend to exhibit improved STL performance in the low-frequency region, where structural rigidity primarily governs sound transmission behavior.

Table 9 summarizes the relative acoustic responses of the tested mixtures based on the NRC values and observed STL ranges. In general, increasing the lightweight aggregate content improved transmission attenuation, particularly in mixtures containing expanded perlite. The PRC-1.0 mixture exhibited the highest NRC value and the widest STL range among the tested specimens.

Overall, the results indicate that lightweight aggregate incorporation may enhance acoustic performance, although the balance between sound response and mechanical strength should also be considered. It should be noted that the acoustic measurements performed in the present study were based on laboratory-scale impedance tube testing under normal-incidence conditions. Therefore, the obtained STL and NRC values may differ from large-scale field applications and diffuse-field acoustic conditions.

## 4. Conclusions

Based on the comprehensive mechanical and acoustic evaluations conducted in this study, including impedance tube measurements and frequency-dependent sound transmission loss analyses, the following conclusions can be drawn:

1. The incorporation of pumice and expanded perlite aggregates significantly influenced the physical and mechanical properties of the lightweight cementitious composites. Increasing the lightweight aggregate replacement ratio generally reduced the compressive strength, flexural strength, modulus of elasticity, impact resistance, and ultrasonic pulse velocity (UPV).
2. The incorporation of lightweight aggregates at high replacement ratios resulted in reductions in compressive and flexural strength. For example, the 28-day compressive strength decreased from 60.12 MPa in the reference mixture to 40.47 MPa in the PMC-1.0 mixture and 47.18 MPa in the PRC-1.0 mixture. This reduction may be attributed to increased porosity and weaker aggregate–paste interaction within the cementitious matrix.
3. The PRC-0.25 mixture exhibited the most balanced overall performance by maintaining relatively high compressive and flexural strength while also providing improved acoustic insulation behavior.
4. Both pumice- and perlite-based mixtures showed improved acoustic responses compared with the reference mixture, particularly at higher replacement levels.
5. Among the tested specimens, the expanded perlite series exhibited more favorable overall acoustic performance. In particular, the PRC-1.0 mixture achieved the highest NRC value and the widest observed STL range.
6. The results indicate a clear trade-off between mechanical strength and acoustic efficiency, where increased porosity and reduced density enhanced sound absorption and transmission attenuation, while reducing compressive strength.
7. Overall, lightweight cementitious composites containing expanded perlite appear to be promising candidates for building applications requiring reduced weight and improved acoustic performance.

The findings obtained in this study are limited to the investigated lightweight aggregate types, replacement ratios, specimen dimensions, and laboratory-scale acoustic measurement conditions. Therefore, the reported mechanical and acoustic performance trends should be interpreted within the scope of the present experimental program. Future studies involving large-scale structural elements, different curing conditions, and detailed microstructural characterization are recommended to further validate the observed behavior. In addition, the absence of direct microstructural characterization and large-scale acoustic testing may be considered among the limitations of the present study. Future research may focus on SEM-based pore structure analysis, long-term durability behavior, and full-scale acoustic insulation performance of lightweight cementitious composites.

## Figures and Tables

**Table 1:** Review on impedance tube tests and NRC results of different lightweight concrete samples.

Authors	Type of Specimen	Materials Used	Density (kg/m^3^)	NRC
Zhao et al. [18]	Lightweight aggregate concrete (LWAC)	Expanded perlite	-	0.54
		Slag	-	0.47
		Clay ceramsite	-	0.45
Sukontasukkul [19]	Lightweight aggregate concrete (LWAC)	10% crumb rubber substituted	2170	0.18
		20% crumb rubber substituted	2110	0.18
Mahmoud et al. [20]	Lightweight aggregate concrete (LWAC)	100% Eps was substituted for dolomite	1500	0.48 (α = 1000 Hz)
		100% expanded clay substituted for dolomite	1812	0.8 (α = 1000 Hz)
		100% vermiculite substituted for dolomite	1798	0.55 (α = 1000 Hz)
Medina et al. [21]	Lightweight aggregate concrete (LWAC)	20% crumb rubber substituted	2264	0.05
		40% crumb rubber substituted	2156	0.04
		60% crumb rubber substituted	2026	0.06
		80% crumb rubber substituted	1858	0.05
Tiwari et al. [22]	Lightweight aggregate concrete (LWAC)	10% cenosphere substituted	1800	0.10
		20% cenosphere substituted	1750	0.12
		30% cenosphere substituted	1690	0.12
		40% cenosphere substituted	1490	0.15
Kapicová et al. [23]	Lightweight aggregate concrete (LWAC)	5% microsilica substituted for cement	1507	0.55
		10% microsilica substituted for cement	1665	0.55
		15% microsilica substituted for cement	1689	0.50
Ling et al. [24]	Lightweight aggregate concrete (LWAC)	30% crumb rubber substituted	-	~0.12
Mohammed et al. [25]	Lightweight aggregate concrete (LWAC)	10% crumb rubber substituted	1870	~0.06
		25% crumb rubber substituted	1700	~0.08
		50% crumb rubber substituted	1510	~0.11
Ngohpok et al. [26]	Lightweight aggregate concrete (LWAC)	40% recycled concrete aggregate substituted	1881	0.38 (SAA)
		60% recycled concrete aggregate substituted	1808	0.36 (SAA)
		100% recycled concrete aggregate substituted	1739	0.38 (SAA)
Zhang et al. [27]	Alkali-activated lightweight concrete	5% foam dosage substituted	1050	~0.10
		10% foam dosage substituted	960	~0.12
Mastali et al. [28]	Alkali-activated lightweight concrete	25% foam dosage substituted	820	0.36
		30% foam dosage substituted	720	0.42
		35% foam dosage substituted	600	0.41
Alyousef [29]	Textile-reinforced lightweight concrete	0.5% wool fibers substituted	-	0.18
		1% wool fibers substituted	-	0.26
		1.5% wool fibers substituted	-	0.32
		2% wool fibers substituted	-	0.35
		2.5% wool fibers substituted	-	0.40
Alyousef [29]	Textile-reinforced lightweight concrete	0.5% salt-treated wool fibres substituted	-	0.21
		1% salt-treated wool fibres substituted	-	0.28
		1.5% salt-treated wool fibres substituted	-	0.35
		2% salt-treated wool fibres substituted	-	0.38
		2.5% salt-treated wool fibres substituted	-	0.42
Chen et al. [30]	Textile-reinforced lightweight concrete	10% miscanthus fibres substituted	1504	0.15
		20% miscanthus fibres substituted	1406	0.11

**Table 2:** Physical properties of cement.

Properties	Analysis Results
Specific gravity (g/cm^3^)	3.15
Initial setting time (min)	146
Final setting time (min)	193
Volume expansion (mm)	1
Specific surface (cm^2^/g)	3730
45 μm sieve residue (%)	5.2
90 μm sieve residue (%)	0.2

**Table 3:** Properties of silica sand.

Experiments	Experiment Result
AFS	28
% Clay	0.19
% SiO_2_	98.71
% Fe_2_O_3_	0.07
% Al_2_O_3_	0.57
Sintering Temperature	>1500 °C
pH	6.85

**Table 4:** Physical properties of the pumice aggregate.

Unit Volume Weight (kg/m^3^)	Water Absorption Capacity (24 h)
389.1	41.40

**Table 5:** Physical and chemical properties of the expanded perlite aggregate.

Property	Value
Physical state	Solid
Appearance and odor	Gray, odorless
Melting point	>1100 °C
Specific gravity	2300 kg/m^3^
Bulk density	1500 kg/m^3^
pH	Neutral

**Table 6:** Properties of the superplasticizer.

Property	Value	Test Method/Conditions
Appearance	Light yellow	Visual
Concentration	Min. 50%	Master solution
pH	5.7	Hydrometer, 25 °C
Specific gravity	1.10	60 rpm
Viscosity	Max. 420 cps	25 °C

**Table 7:** Mix designs.

Sample Code	Ratio	W/C Ratio	Cement	Water	Plasticizer	Silica Sand	Pumice	Expanded Perlite
Reference	0	0.4	550	220	5.5	1559.46	--	--
PMC-0.25	0.25					1169.60	202.15	--
PMC-0.5	0.50					779.733	404.30	--
PMC-0.75	0.75					389.86	606.45	--
PMC-1.0	1.00					--	808.61	--
PRC-0.25	0.25					1169.60	--	194.93
PRC-0.5	0.50					779.73	--	389.86
PRC-0.75	0.75					389.86	--	584.80
PRC-1.0	1.00					--	--	779.73

**Table 8:** Flow table test results.

Series	Spread Diameter (cm)
Reference	12.50
PMC-0.25	13.75
PMC-0.50	14.50
PMC-0.75	16.75
PMC-1.0	18.40
PRC-0.25	28.40
PRC-0.50	30.10
PRC-0.75	32.25
PRC-1.0	33.60

**Table 9:** Comparative summary of the acoustic responses of the tested lightweight cementitious composites based on the NRC values and observed STL ranges.

Mixture Code	NRC	Maximum STL Range	Acoustic Behavior
REF	0.35	22–25 dB	Baseline performance
PMC-0.25	0.31	20–24 dB	Similar to reference
PMC-0.50	0.30	22–26 dB	Moderate attenuation
PMC-0.75	0.19	26–30 dB	High-frequency improvement
PMC-1.0	0.28	28–31 dB	Better broadband response
PRC-0.25	0.31	21–24 dB	Stable response
PRC-0.50	0.25	24–27 dB	Mid-frequency enhancement
PRC-0.75	0.20	27–31 dB	Higher attenuation
PRC-1.0	0.47	30–34 dB	Best overall acoustic performance

## Data Availability

The raw data supporting the conclusions of this article will be made available by the authors on request.
